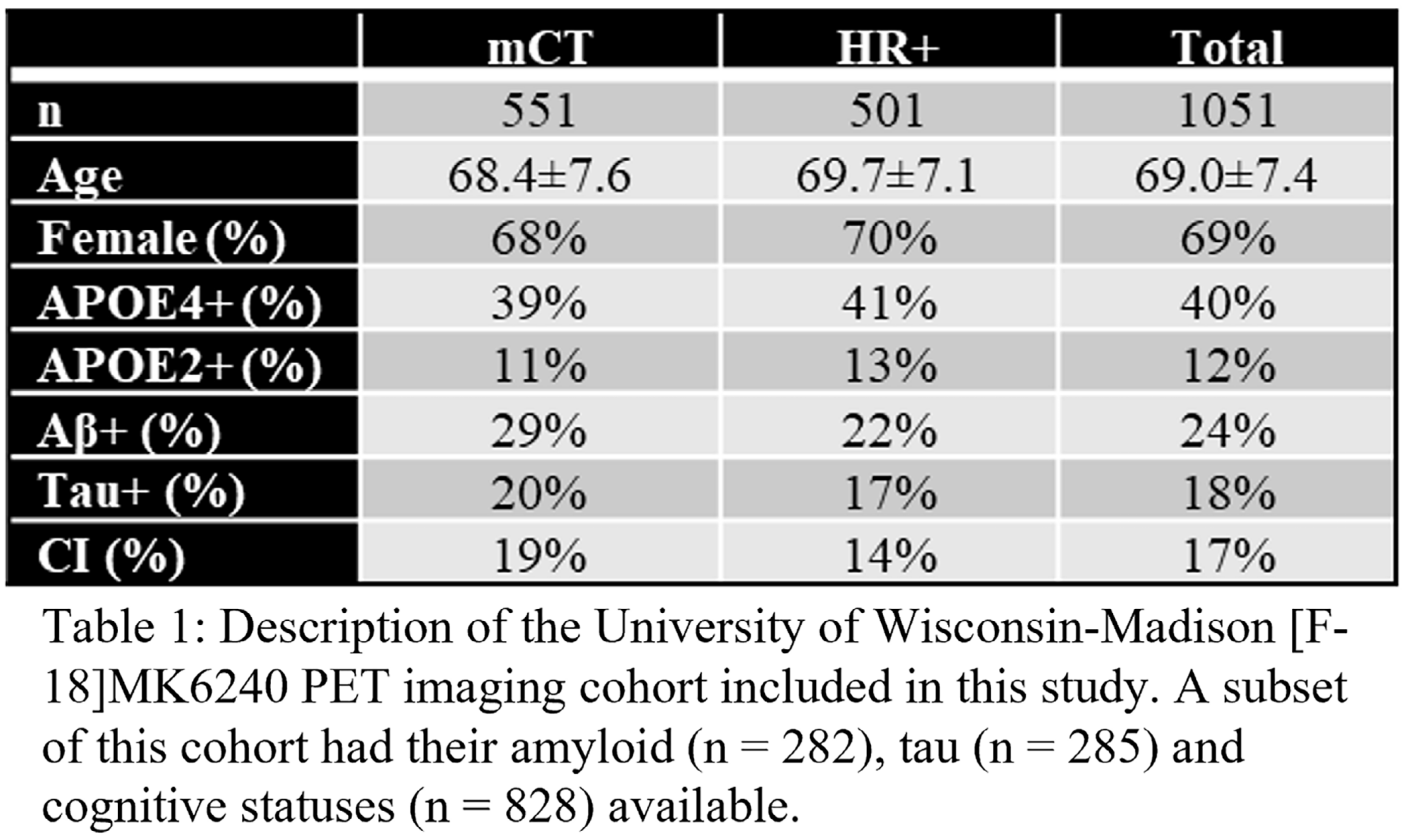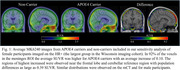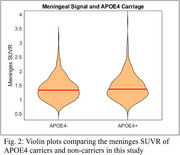# Higher off‐target meninges [F‐18]MK6240 PET signal associated with APOE4 carriage

**DOI:** 10.1002/alz70856_099910

**Published:** 2025-12-25

**Authors:** Andrew K McVea, Max McLachlan, Brecca Bettcher, Dana L Tudorascu, Theresa M. Harrison, Susan M. Landau, Sterling C Johnson, Matthew D Zammit, Tobey J. Betthauser, Bradley T Christian

**Affiliations:** ^1^ University of Wisconsin ‐ Madison, Madison, WI, USA; ^2^ Waisman Center, University of Wisconsin‐Madison, Madison, WI, USA; ^3^ University of Pittsburgh, Pittsburgh, PA, USA; ^4^ Neuroscience Department, University of California, Berkeley, Berkeley, CA, USA; ^5^ Department of Medicine, University of Wisconsin‐Madison School of Medicine and Public Health, Madison, WI, USA; ^6^ University of Wisconsin‐Madison School of Medicine and Public Health, Madison, WI, USA

## Abstract

**Background:**

Individuals with the APOE4 allele have a lifetime enhanced risk for Alzheimer's disease (AD) including earlier average onset of amyloid and tau. [F‐18]MK6240 is a PET radioligand that binds to tau aggregates in AD, however, variable MK6240 off‐target signal in the meninges adjacent to target regions can influence PET quantification. Previous studies (Smith, 2021) have identified higher MK6240 meninges signal in females and a PET signal dependence on scanner model. The goal of this study is to compare the magnitude and distribution of meninges MK6240 signal observed in APOE4 carrier and non‐carrier populations.

**Method:**

All participants (*n* = 1051) were scanned at the University of Wisconsin–Madison from 90‐110 minutes on a Biograph Horizon mCT or ECAT HR+ (Table 1). MK6240 PET images were processed using a standardized pipeline to generate SUVR images using the inferior cerebellar grey matter reference region and smoothed to a common 6mm resolution. The meninges ROI used was created by diluting the MNI‐152 cortical brain mask by 5mm and then subtracting the original mask. This mask was then warped into native space for analysis. APOE4 carriers and non‐carriers were compared using a multiple regression model inlcuding meninges SUVR with APOE4 carriage, sex, scanner model and the interaction terms between variables. In a subgroup of the study a sensitivity analysis including female participants imaged on the HR+ (*n* = 349) was performed with a student's t‐test comparing carriers and non‐carriers.

**Result:**

Higher average meninges signal was observed for APOE4 carriers (*p* = 0.03), females (*p* < 0.001) and participants imaged on the mCT (*p* < 0.001). No significant interaction terms were observed. In the sensitivity analysis female APOE4 carriers on the HR+ had significantly higher meninges signal (*p* = 0.009). Similar results were observed with female participants on the mCT (*p* = 0.05), although the differences for males were not significant on either scanner (*p* = 0.24, *p* =  0.15).

**Conclusions:**

Spill‐in effects from MK6240 meninges signal can potentially bias tracer outcomes in target analysis regions. Meninges signal can be highly variable, but the relationships between APOE4 carriage and sex on this measure should be accounted for in MK6240 quantification and population‐based comparisons.